# Tracking Chromosome Evolution in Southern African Gerbils Using Flow-Sorted Chromosome Paints

**DOI:** 10.1159/000350696

**Published:** 2013-05-04

**Authors:** L.I. Knight, B.L. Ng, W. Cheng, B. Fu, F. Yang, R.V. Rambau

**Affiliations:** ^a^Evolutionary Genomics Group, Department of Botany and Zoology, University of Stellenbosch, Stellenbosch, South Africa; ^b^Wellcome Trust Sanger Institute, Wellcome Trust Genome Campus, Hinxton, UK

**Keywords:** Chromosome painting, Gerbillinae, *Gerbilliscus paeba*, Robertsonian rearrangements

## Abstract

*Desmodillus* and *Gerbilliscus* (formerly *Tatera*) comprise a monophyletic group of gerbils (subfamily Gerbillinae) which last shared an ancestor approximately 8 million years ago; diploid chromosome number variation among the species ranges from 2n = 36 to 2n = 50. In an attempt to shed more light on chromosome evolution and speciation in these rodents, we compared the karyotypes of 7 species, representing 3 genera, based on homology data revealed by chromosome painting with probes derived from flow-sorted chromosomes of the hairy footed gerbil, *Gerbillurus paeba* (2n = 36). The fluorescent in situ hybridization data revealed remarkable genome conservation: these species share a high proportion of conserved chromosomes, and differences are due to 10 Robertsonian (Rb) rearrangements (3 autapomorphies, 3 synapomorphies and 4 hemiplasies/homoplasies). Our data suggest that chromosome evolution in *Desmodillus* occurred at a rate of ∼1.25 rearrangements per million years (Myr), and that the rate among *Gerbilliscus* over a time period spanning 8 Myr is also ∼1.25 rearrangements/Myr. The recently diverged *Gerbillurus (G. tytonis* and *G. paeba)* share an identical karyotype, while *Gerbilliscus kempi, G. afra* and *G. leucogaster* differ by 6 Rb rearrangements (a rate of ∼1 rearrangement/Myr). Thus, our data suggests a very slow rate of chromosomal evolution in Southern African gerbils.

Southern African gerbils are a monophyletic assemblage of rodents composed of 3 genera and 9 species occurring predominantly in the arid parts of this subregion. They include the monotypic *Desmodillus (Desmodillus auricularis)* and the polytypic genera *Gerbilliscus (G. afra, G. leucogaster, G. brantsii, G. inclusa)* and *Gerbillurus (G. paeba, G. tytonis, G. vallinus, G. setzeri)* [Chimimba and Bennett, 2005; Musser and Carleton, 2005]. The radiation of Gerbillinae spans roughly 10 million years (Myr) with one of the most derived species among Southern African taxa *(G. paeba)* having diverged ∼2.9 million years ago (Mya) [Chevret and Dobigny, 2005; Colangelo et al., 2007]. Our view of evolutionary relationships among the taxa has fluctuated significantly over the past 2 decades. Initially, *Gerbilliscus* and *Gerbillurus* were treated as separate genera [Pavlinov, 2001; Chimimba and Bennett, 2005; Musser and Carleton, 2005]. However, mitochondrial and nuclear sequence data indicate that some of the lineages within *Gerbilliscus* cluster with *Gerbillurus* [Chevret and Dobigny, 2005; Colangelo et al., 2007; Granjon et al., 2012], suggesting a polyphyletic origin for the genera, which in turn prompted the recommendation for *Gerbillurus* to be considered a synonym of *Gerbilliscus*. Here, we adopt the name *Gerbilliscus* (subsuming *Gerbillurus*) in recognition of the patterns suggested by the robust molecular phylogenies [Colangelo et al., 2007; Granjon et al., 2012]. In effect, this implies that *Gerbilliscus* contains 3 lineages: an eastern group *(G. robusta, G. phillipsi, G. vicinus, G. nigricaudus)*, a western group *(G. gambianus, G. guinea, G. kempi)*, and a southern group *(G. brantsii, G. afra, G. leucogaster)*, with *G. paeba*, *G. setzeri* and *G. vallinus* basal to the western + southern clade.

Diploid chromosome numbers of Southern African gerbils range from 2n = 36 *(G. paeba)* to 2n = 50 *(D. auricularis)* [Qumsiyeh, 1986a, b; Qumsiyeh and Chesser, 1988; present data], and G-band analyses indicate that the variation is due primarily to Robertsonian (Rb) fusions and inversions, with the exception of *G. paeba* and *G. tytonis* which have nearly identical karyotypes [Qumsiyeh et al., 1991; Colangelo et al., 2007]. Intrageneric rearrangements among *G. leucogaster, G. afra* and *G. kempi* involve maximally 3 Rb fissions and 3 Rb fusions [Qumsiyeh, 1986b; Volobouev et al., 2007]. Although phylogenetic inferences based on chromosome banding data are valuable [Kovalskaya et al., 2011; Sannier et al., 2011], unequivocal establishment of orthology across taxa may be problematic, and this may hamper detailed descriptions of chromosome rearrangements driving chromosome evolution within taxonomic groups.

Here genome-wide comparisons of the Southern African *D. auricularis, G. tytonis, G. leucogaster, G. afra*, the West African *G. kempi* and the North African *Psammomys obesus* were undertaken in order to refine previous comparisons based on G-banding. In doing so, we aim at providing new insights into chromosomal rearrangements that distinguish these Southern African taxa. This was accomplished using *G. paeba* whole-chromosome painting probes (reported here for the first time) to identify regions of orthology, and to document genome repatterning among *Desmodillus (D. auricularis), Gerbilliscus (G. paeba, G. tytonis, G. leucogaster, G. afra, G. kempi)* and the outgroup species, *P. obesus*. Interpreting these differences in the context of the robust DNA sequence-based phylogeny [Chevret and Dobigny, 2005; Colangelo et al., 2005, 2007; Granjon et al., 2012] allowed inferences on the mode and rate of chromosome evolution in Southern African gerbils.

## Materials and Methods

### Specimens and Tissue Culture

Seven species from 3 gerbilline genera were examined. These include 5 Southern African taxa *(D. auricularis, G. paeba, G. tytonis, G. leucogaster*, and *G. afra)* as well as *G. kempi* (West Africa) and *P. obesus* (North Africa), respectively (table 1). Tissue biopsies (tail or rib muscle) were used to establish fibroblast cultures in DMEM medium (GIBCO) enriched with 15% foetal calf serum (FCS; GIBCO), amniomax supplement with amniomax basal medium (GIBCO) and Gentamicin (50 µg/ml). Cell cultures were incubated in chambers regulated at 37°C and 5% CO_2_. Cell division was arrested at metaphase stage using colcemid (with final concentration at 0.10 µg/ml; GIBCO) and metaphase cells were harvested using a 0.075 M KCl hypotonic treatment and subsequent fixation with modified Carnoy's fixative (methanol:glacial acetic acid, 3:1).

### Chromosome Banding and Karyotypes

Chromosome G- and C-banding followed the protocols of Seabright [1971] and Sumner [1972], respectively. Briefly, G-banding of metaphase chromosome spreads required aging the slides for a minimum of 1 h at 65°C and treating aged slides with a trypsin solution (0.25% in 1× PBS) for a minimum of 30 s. Trypsin-treated slides were rinsed in calf serum buffer (containing 500 µl FCS and 50 ml 0.025 M KH_2_PO_4_ buffer, pH 6.8) for 3 min followed by Giemsa staining (made in 0.025 M KH_2_PO_4_ buffer; pH 6.8) to visualise chromosomes. C-banding entailed immersing slides in a 0.2 M HCl solution for 3 min, followed by incubation in a saturated Ba(OH)_2_ solution for a minimum of 70 s at 55°C. A second incubation in 2× SSC for 30-60 min at 50-60°C (species-dependent) followed. Chromosomes were visualised using 2% Giemsa staining and arranged according to karyotypes published by Qumsiyeh [1986a, b], Qumsiyeh and Chesser [1988] and Volobouev et al. [2007].

### Flow-Sorting and Fluorescent in situ Hybridization

*G. paeba* (2n = 36) was selected for flow-sorting primarily because it is a terminal taxon among the Southern African species in the molecular phylogeny [see Chevret and Dobigny, 2005]. Chromosomes were sorted on a MoFlo dual-laser cell sorter and isolated on size and base-pair composition [Yang et al., 2009]. Biotin-16-dUTP- (Roche) or digoxigenin-11-dUTP-labelled painting probes were made by DOP-PCR amplification of flow-sorted chromosomes [Telenius et al., 1992]. Fluorescent in situ hybridization (FISH) experiments followed Rens et al. [2006] and Yang and Graphodatsky [2009] with minor modifications. Biotin-labelled probes were detected with the antibody Cy3-streptavidin (1:500 dilution), and digoxigenin-labelled probes with FITC-conjugated anti-digoxigenin IgG made in sheep (1:1,000). Slides were counterstained with 0.1 µg/ml DAPI (4′,6-diamidino-2-phenylindole) and mounted using Vectashield medium (Vector Labs). Images were captured using a CCD camera attached to an Olympus BX60 epifluorescence microscope equipped with DAPI, FITC and Cy3 fluorescence filters. Images were edited using the Genus software system (Applied Imaging Corp., Newcastle, UK).

### Consensus Phylogeny

A consensus phylogenetic tree that includes the species under examination (i.e. *D. auricularis, G. paeba, G. tytonis, G. leucogaster, G. afra, G. kempi*, and *P. obesus*) was obtained from the more comprehensive DNA-based phylogenies of Dobigny and Chevret [2005], Colangelo et al. [2005, 2007] and Granjon et al. [2012]. In effect, our tree emphasises the monophyly of *Gerbilliscus + Gerbillurus* (both referred to as *Gerbilliscus*), the basal position of *Desmodillus* to the Southern African taxa, and the clustering of *P. obesus* outside the Southern African taxa, all well-established evolutionary associations. The latter species was used to polarize some of the chromosomal changes observed at the base of Southern African taxa.

## Results and Discussion

Characterisation of the G. paeba Flow Karyotype and Cross-Species FISH

**Fig. 1:**
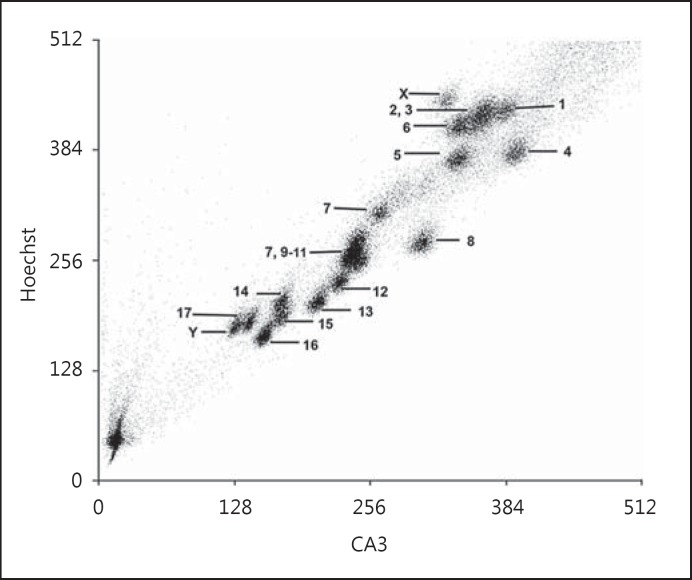
Bivariate flow karyotype of a male fibroblast cell line from *G. paeba* (2n = 36).

The 36 chromosomes of *G. paeba* (GPA; 2n = 36) were resolved into 16 peaks, of which 14 each contained a single type of autosome (1, 4-6, 7-8, 12-17, X, Y), 1 peak contained 2 autosomes (2 + 3) and 1 contained 4 autosomes (7 + 9 + 10 + 11; fig. 1). The homologues of GPA7, which are C-positive, were sorted into 2 peaks, likely as a result of variation in the amount of heterochromatin.

**Fig. 2:**
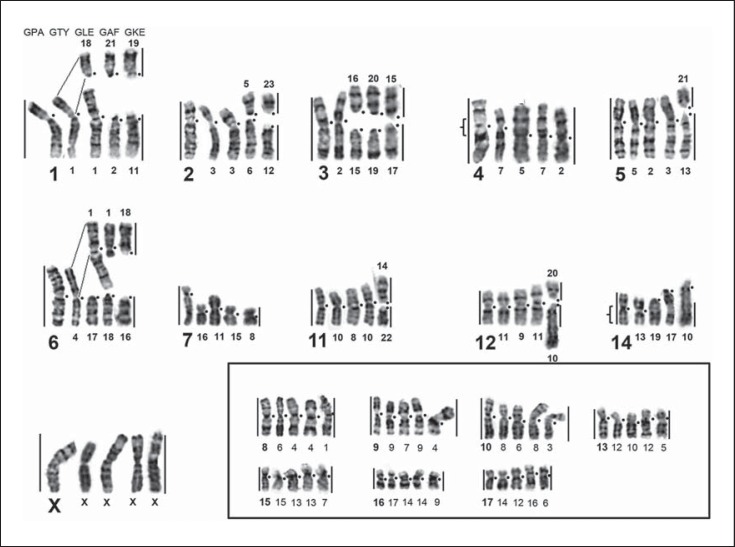
Partial homology map among *G. paeba* (GPA), *G. tytonis* (GTY), *G. leucogaster* (GLE), *G. afra* (GAF), and *G. kempi* (GKE) based on chromosome painting and G-banding, with GPA chromosomes as references. Chromosomes that were conserved in their entirety in all *Gerbilliscus* taxa are in the inset. The regions of homology are indicated by the vertical lines and GPA chromosome numbers (large font). Centromere positions are indicated by closed circles, and the brackets indicate the boundaries of inversions. Chromosome numbers correspond to those in the respective species' karyotypes.

**Fig. 3:**
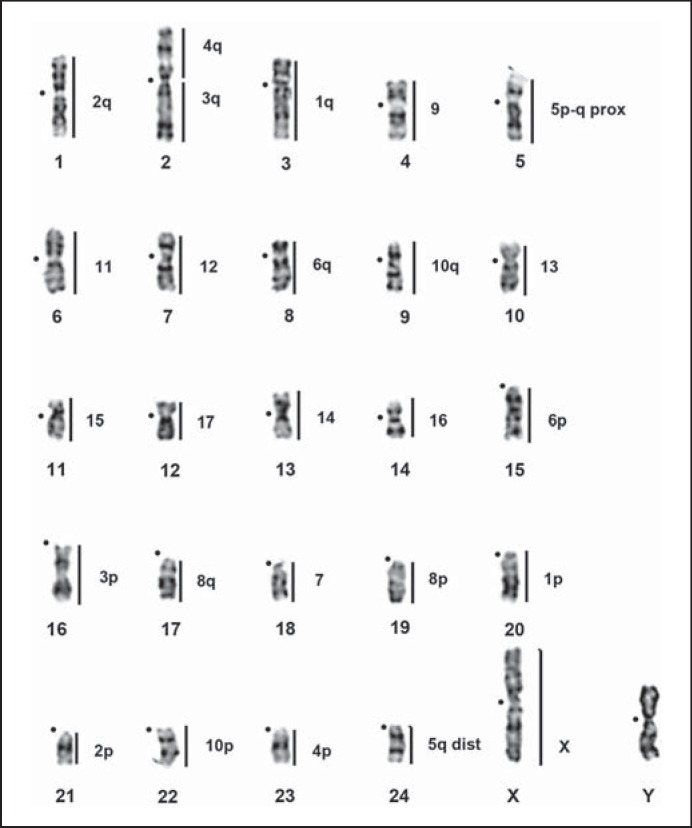
G-banded haploid karyotype of a male *D. auricularis* with regions of orthology to *G. paeba* indicated by vertical lines (numbered to the right) as determined by cross-species chromosome painting. Centromere positions are indicated by closed circles.

**Fig. 4:**
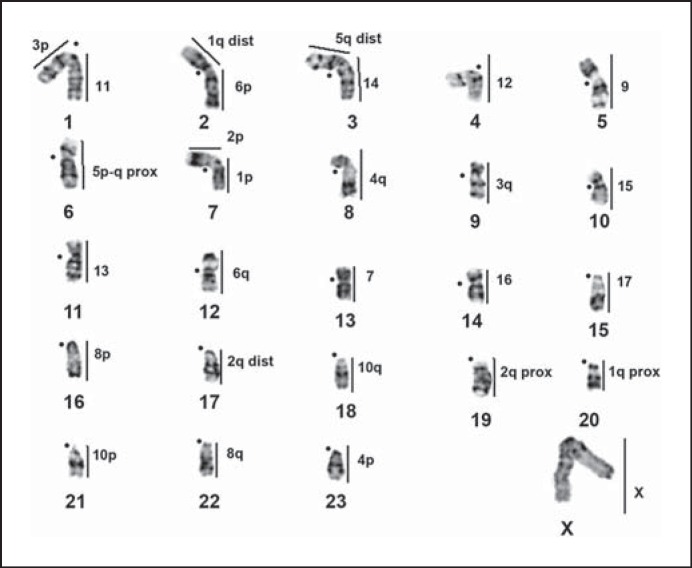
G-banded haploid karyotype of a male *P. obesus* with regions of orthology to *G. paeba* indicated by vertical lines (numbered to the right) as determined by cross-species chromosome painting. Centromere positions are indicated by closed circles.

**Fig. 5:**
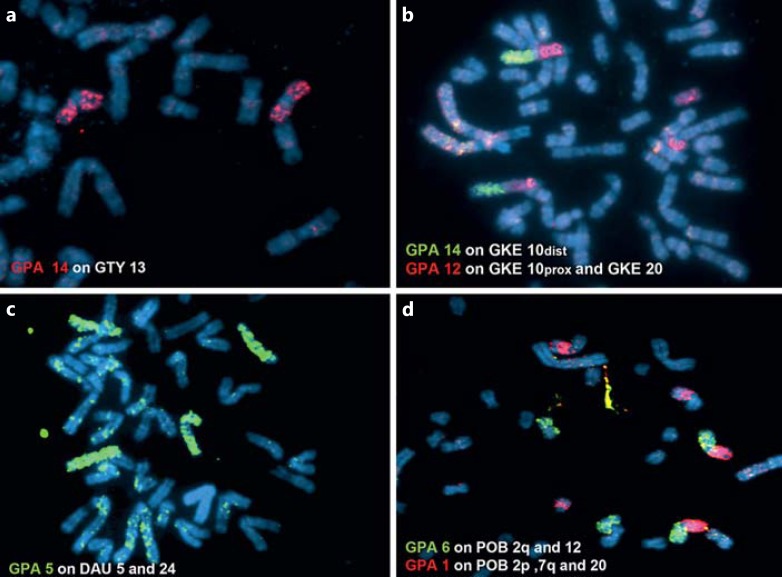
Examples of FISH of *G. paeba* (GPA) painting probes onto *G. tytonis* (GTY), *G. kempi* (GKE)*, D. auricularis* (DAU), and *P. obesus* (POB). **a** Hybridization of GPA14 (Cy3) on GTY13. **b** Hybridization of GPA12 and GPA14 on GKE10proximal and 20, and GKE10distal, respectively. **c** FISH of GPA5 on DAU5 and 24. **d** FISH of GPA1 and GPA6 on POB2p,7q, and 20 and POB2q and 12, respectively. Chromosomes are counterstained with DAPI.

*G. paeba* chromosome paints were successfully hybridized onto metaphase chromosomes of all gerbil taxa analyzed, enabling detailed comparisons among them (figs. 2, 3, 4, 5; table 2). Together with banding comparisons, the data from chromosome painting show slight differences in genome conservation among the species. Nineteen homologous regions were identified in *G. tytonis* (GTY; fig. 2) – all chromosomes were conserved intact (correspondence of the probe containing multiple chromosomes was verified using G-bands of *G. paeba*). The same set of chromosome paints delimited 21 synteny-conserved regions in *G. leucogaster* (GLE), 22 regions in *G. afra* (GAF) (fig. 2) and 25 in *G. kempi* (GKE) (fig. 2). In these 3 species *(G. leucogaster, G. afra, G. kempi)*, 14 to 15 GPA chromosomes (GPA4-17 and the X) are each retained as a single chromosome, and 3 GPA chromosomes (GPA1, 3, 6) each correspond to 2 chromosomes. Furthermore, GPA2 probe painted 2 segments in *G. afra* and *G. kempi,* while GPA5 and 12 produced 2 signals in *G. kempi* (fig. 2). Cross-species chromosome painting onto metaphases of *D. auricularis* (DAU) revealed 26 regions of homology (fig. 3). Of these, 10 were conserved as single chromosomes homologous to GPA7, 9, 11-17 and the X, and 8 were conserved as 2 chromosomes, or chromosome segments, corresponding to GPA1-6, 8 and 10 (fig. 3). Lastly, 28 homologous regions were detected in *P. obesus* (POB), wherein 10 regions each correspond to 1 GPA chromosome (GPA7, 9, 11-17 and X), 6 GPA chromosomes (GPA3-6, 8 and 10) each are homologous to 2 POB chromosomal fragments, and 2 (GPA1 and 2) each are homologous to 3 POB fragments (table 2; fig. 4). The level of genome conservation is consistent with previously established evolutionary relations among these species: *Desmodillus* is basal in the phylogeny, while the *Gerbilliscus* taxa form a monophyletic clade with *G. paeba* and *G. tytonis* possessing identical karyotypes [Chevret and Dobigny, 2005; Colangelo et al., 2007; Granjon et al., 2012].

### Discrepancies between G-Banding and FISH Results

While the FISH results were largely in agreement with published data based on G-band comparison [Qumsiyeh, 1986a, b; Qumsiyeh and Chesser, 1988; Qumsiyeh et al., 1991; Colangelo et al., 2001; Volobouev et al., 2007], we were able to correct several mismatches in the identification of presumptive homologues and resolve some of the problematic associations reported in previous comparisons. This is most evident in the comparison between *G. kempi* and *G. tytonis* [Volobouev et al., 2007] as well as between *Desmodillus* and *G. paeba* [Qumsiyeh, 1986a, b]. Banding analyses initially suggested GTY1, 2, 3, 5, 10 and 11 to be homologous to GKE17 + 11, 15 + 14, 12 + 20, 13 + 19, 21 + 22 and 23 + 10prox, respectively [Volobouev et al., 2007]. Our data unequivocally identified GTY1 = GKE11 + 19, GTY2 = GKE15 + 17, GTY3 = 12 + 23, GTY5 = GKE13 + 21, GTY10 = GKE14 + 22, and GTY11 = GKE20 + 10prox (fig. 2). Secondly, DAU2 was initially described as homologous to GPA3q plus a de novo euchromatic addition [Qumsiyeh, 1986b]. Our results indicate that the ‘euchromatic addition’ is in fact homologous to GPA4q (fig. 3).

Further, the so-called late replicating GPA7 [Qumsiyeh et al., 1991] successfully hybridized to all species analysed here and was retained as a single chromosome in all species (figs. 2, 3, 4). The differences among the chromosomes painted by GPA7 are their sizes, and, in one instance in *Desmodillus* (DAU18, fig. 3), the difference is due to a pericentric inversion resulting in an acrocentric form (in contrast, all GPA7 homologues in other gerbils are biarmed). Although previous banding comparison suggested GPA7 is wholly heterochromatic [Qumsiyeh, 1988; Qumsiyeh et al., 1991], our data refutes this, especially given the fact that it successfully hybridized to euchromatic genomic fractions in all related species, taxa that last shared an ancestor approximately 9 Mya [Chevret and Dobigny, 2005].

Cross-species chromosome painting also revealed a striking resemblance between karyotypes of *G. paeba* and *G. tytonis,* confirming previous banding studies. All GPA chromosomes, including the Y chromosome, were retained in *G. tytonis*. In addition, the 2 species are very similar in other respects, notwithstanding their sympatric distribution in Namibia. Other aspects identical between the 2 species include quadrupedal saltation (adaptation for locomotion in sand), absence of sexual dimorphism, nocturnal activity and omnivorous diet [Griffin, 1990; Perrin et al., 1999a, b]. The overlap between the taxa is considerable. For example, *G. paeba* from the Namib Desert weighs on average 26.40 g (20-37 g), has a slightly tufted tail tip, and the hind foot is less than 30 mm (21-30 mm), while *G. tytonis* has an average mass of 24 g (maximum 35 g), a more pronounced, tufted tail, and narrow, hairy feet with hind foot length averaging 33.3 mm (28-36 mm) [Griffin, 1990; Perrin et al., 1999a, b; Chimimba and Bennett, 2005]. Such a pronounced overlap in morphology and habitat preference coupled to their invariant karyotypes suggests a recent divergence and further investigations may show them to be the same species.

### Chromosomal Rearrangements and Homoplasy

**Fig. 6:**
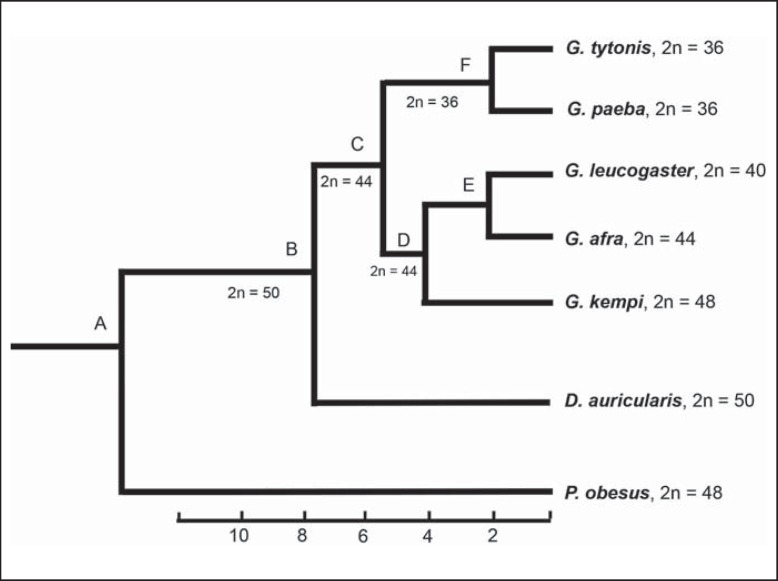
A consensus tree derived from mtDNA and nuclear sequence data [Chevret and Dobigny, 2005; Colangelo et al., 2005, 2007; Granjon et al., 2012]. The diploid numbers and the approximate divergence dates were obtained from Colangelo et al. [2007]. Node A is characterised by gross chromosomal homeologies in all species identified with GPA7, 9, 13, 15-17, and the syntenic association GPA4q/3q defines split of *D. auricularis* at node B. The nodes C-F contain 10 Robertsonian rearrangements of which 4 (involving GPA1, 2, 3, 5) were characterised as hemiplasies or homoplasies [Avise and Robinson, 2008]. For detailed discussion see text and table 2.

Our chromosomal homology map established by FISH and banding comparison demonstrates that Rb rearrangements were important in chromosomal evolution and speciation in these gerbils (figs. 2, 6). Early G-band comparisons among gerbils have suggested that Rb rearrangements are homoplasic, and interpreting them in an evolutionary context may be problematic [Qumsiyeh et al., 1987] – a finding recently shown for Bovidae [Robinson and Ropiquet, 2011]. Using the gerbil sequence-based phylogeny as scaffold (fig. 6), we show that of the 10 rearrangements (table 2) identified by the GPA painting probes 1-6, 8, 10, 11, and 12, one is an asynapomorphy (GPA6) uniting *G. paeba* + *G. tytonis*, 2 are autapomorphies (GPA11 and 12) in *G. kempi*, 3 are synapomorphies (GPA4, 8 and 10) for all *Gerbilliscus*, and the remainder (GPA1, 2, 3, 5) are potential homoplasies/hemiplasies [Avise and Robinson, 2008].

With respect to the first of these, GPA1, 2 possible hypotheses may be suggested for the observed patterns when the character is mapped to the tree in figure 6. The first is that it is an example of hemiplasy, having undergone a fusion at ∼6 Mya (node C) and persisted as a polymorphism until the divergence of *G. leucogaster* and *G. afra* at ∼2.5 Mya. The alternate explanation (i.e. homoplasy) suggests that the fusion arose at node F (the common ancestor to *G. tytonis* and *G. paeba*) and convergently so in *G. leucogaster* (i.e. requiring 2 changes vs. the one for hemiplasy). Although hemiplasy offers the most parsimonious solution in terms of rare genomic changes [Rokas and Holland, 2000], the required persistence time (∼3.5 Myr) is at the upper bound suggested by Robinson et al. [2008] and Robinson and Ropiquet [2011], and we regard homoplasy as the more likely of the 2 hypotheses. The same reasoning applies to the Rb rearrangements involving GPA2 and GPA3. A more convincing case for hemiplasy can be made with respect to GPA5 (arose at node C and persisted as a polymorphism for ∼2 Myr to D, at which point it was lost in the lineage leading to *G. kempi*).

Consequently, the finding of Qumsiyeh et al. [1987] that homoplasy is present in the chromosomal evolution of gerbils is confirmed by the present study. Our results show that 4 of the 10 rearrangements identified are homoplasic when mapped to the tree (3 convergences/reversals and 1 probable hemiplasy, all of which contribute to the ‘noise’ evident in the species phylogeny). Qumsiyeh et al. [1987] on the other hand obtained 7 homoplasies in 15 Rb rearrangements among *G. paeba, G. vallinus, G. robustus, G. nigricauda, G. leucogaster, G. afra*, and *G. brantsii*. It should be noted, however, that the Qumsiyeh et al. [1987] study may have overestimated the number of homoplasic traits due to topological differences in the trees used (allozyme-based in Qumsiyeh et al. [1987], and mtDNA and nuclear sequences in our case) and, in some instances, to their misidentification of the chromosomes involved.

In addition to the overwhelming presence of Rb rearrangements in these gerbils, inversion differences were also noted in several species. Side-by-side G-band comparison (directed by FISH) in *Desmodillus* and *G. afra* show that 5 autosomal pairs differ due to the position of the centromeres (DAU1 vs. GAF6, DAU3 vs. GAF2, DAU13 vs. GAF17, DAU8 vs. GAF18, DAU18 vs. GAF15; figs. 2, 3). Similarly, 2 GPA autosomes differ from their orthologs in *G. afra* as a result of centromere variability (GAF17 vs. GPA14 and GAF7 vs. GPA4; online suppl. fig. 1, www.karger.com/doi/10.1159/000350696). Similar observations have been documented for other gerbil species [Volobouev et al., 2007].

### Ancestral Karyotype Inference

The use of the DNA sequence phylogeny and the inclusion of *P. obesus* in the comparisons allow us to propose an ancestral karyotype for the last common ancestor to *Desmodillus* and *Gerbilliscus.* We hypothesise that this comprised 7 biarmed autosomes which remained unchanged among all the taxa (GPA7, 9, 13, 15, 16, 17, X; table 2), and 21 acrocentrics (GPA1p, 1q, 2p, 2q, 3p, 3q, 4p, 4q, 5p, 5q, 6p, 6q, 8p, 8q, 10p, 10q, 11p, 11q, 12p, 12q and 14). Further refinements to this karyotype can be expected since *Psammomys* is only 1 of 3 legitimate outgroup taxa *(Brachiones, Desmodilliscus, Pachyuromus)* to the Southern African gerbil species.

### Rate of Chromosome Evolution

Interpretation of the rearrangements in the context of DNA sequence divergence times allows us to make inferences about the rate of evolution. *Desmodillus* accumulated 4 Rb rearrangements and 5 inversions since it separated from *Gerbilliscus*, reflecting a rate of 1.25 rearrangements/Myr (even taking the homoplasic rearrangement detected by GPA5 into account); *G. kempi* accumulated 3 Rb rearrangements following its split from the *G. afra/G. leucogaster* lineage 5.5 Mya, which translates into an evolutionary rate of less than 1 rearrangement/Myr. The 2 rearrangements separating *G. leucogaster* from *G. afra* accumulated over 2.5 Mya, representing a rate of approximately 1 rearrangement/Myr. On average, the rate of chromosomal evolution among *Gerbilliscus* over 8 Myr is ∼1.25 rearrangement/Myr (10 rearrangements over 8 Myr), which is considerably slower than values previously obtained in other gerbils (e.g. West African *Taterillus* have a rate of 45 rearrangements/Myr) [Dobigny et al., 2002, 2005]. Some *Gerbillus* species show a rate of ∼12.3 rearrangements/Myr [Aniskin et al., 2006; Chevret and Dobigny, 2005]. Recently, it was shown that 6 representative genera in the Rattini tribe displayed an evolutionary rate of 0.6-3.33 rearrangements/Myr, which was considered slow for murid rodents [Badenhorst et al., 2011]. Therefore, the rate of chromosome evolution in the Southern African gerbils studied here is unexpectedly slow.

In summary, we report the first genome-wide cross-species chromosome painting in Southern African gerbilline rodents. The FISH data unequivocally establish homology among taxa from *Desmodillus* and *Gerbilliscus* which last shared an ancestor approximately 8 Mya. Our data also corrected some errors in published homeology maps that were based on banding results alone, and thus underscores the utility of FISH for inferring homology between species. Significantly, the present data demonstrates that the rate of chromosome evolution in *Gerbilliscus* is relatively slow in comparison to other gerbil lineages, which suggests evolutionary rate heterogeneity within these rodents. A more complete Zoo-FISH analysis including more representatives of the 15 genera comprising the subfamily Gerbillinae should provide more detailed assessment of chromosome evolution among these rodents.

## Supplementary Material

Supplemental Figure

## Figures and Tables

**Table 1:** Specimens examined in the study and their respective localities in South Africa (RSA), Namibia, West and North Africa

Species	Locality	Map coordinates	Sex	2n	Specimen (η)
*Desmodillus auricularis*	Henkries, Northern Cape, RSA	28°57′57″S, 18°08′54″E	♂	50	1
	Tankwa Karoo, Northern Cape, RSA	32°12′19″S, 19°09′10″E	♂	50	2
	Three Sisters, Northern Cape, RSA	31°53′14″S, 23°05′17″E	♂	50	1

*Gerbilliscus kempi*	Senegal	−	♀	48	1

*Gerbilliscus afra*	Clanwilliam, Western Cape, RSA	32°10′42″S, 18°53′27″E	♂	44	1
	Clanwilliam, Western Cape, RSA	32°10′42″S, 18°53′27″E	♀	44	1
	Vensterklip, Western Cape, RSA	32°18′56″S, 18°23′46″E	♀	44	3

*Gerbilliscus leucogaster*	Windhoek, Namibia	22°33′34″S, 17°05′56″E	♂	40	1
	Windhoek, Namibia	22°33′34″S, 17°05′56″E	♀	40	1

*Gerbilliscus paeba*	Anysberg NR, Northern Cape, RSA	33°23′20″S, 20°39′20″E	♂	36	1
	Sutherland, Northern Cape, RSA	33°28′48″S, 20°36′20″E	♂	36	3
	Swakopmund, Namibia	22°40′36″S, 14°31′35″E	♂	36	1
	Swakopmund, Namibia	22°40′36″S, 14°31′35″E	♀	36	1
	Three Sisters, Northern Cape, RSA	31°53′14″S, 23°05′17″E	♂	36	1

*Gerbilliscus tytonis*	Swakopmund, Namibia	22°40′36″S, 14°31′35″E	♂	36	1
	Swakopmund, Namibia	22°40′36″S, 14°31′35″E	♀	36	1

*Psammomys obesus*	Tunisia	−	♀	48	1

**Table 2:** Presence (+) and absence (−) of Robertsonian rearrangements in the 7 gerbil species analysed

Taxa	Rb fissions/fusions*									
	GPA1*	GPA2*	GPA3*	GPA4*	GPA5*	GPA6*	GPA8*	GPA10*	GPA11	GPA12
*P. obesus*	–	–	–	–	–	–	–	–	–	–
*D. auricularis*	–	–	–	–	–	–	–	–	–	–
*G. kempt*	–	–	–	+	–	–	+	+	+	+
*G. afra*	–	–	–	+	+	–	+	+	–	–
*G. leucogaster*	+	+	–	+	+	–	+	+	–	–
*G. tytonis*	+	+	+	+	+	+	+	+	–	–
*G. paeba*	+	+	+	+	+	+	+	+	–	–

Chromosome numbers correspond to *G. paeba* (GPA), the species used for flow-sorting the painting probes. Robertsonian fusions are indicated by an asterisk.

## References

[B1] Aniskin VM, Benazzou T, Biltueva L, Dobigny G, Granjon L, Volobouev V (2006). Unusually extensive karyotype reorganization in four congeneric *Gerbillus* species (Muridae: Gerbillinae). Cytogenet Genome Res.

[B2] Avise JC, Robinson TJ (2008). Hemiplasy: a new term in the lexicon of phylogenetics. Syst Biol.

[B3] Badenhorst D, Dobigny G, Adega F, Chaves R, O'Brien PCM (2011). Chromosomal evolution in Rattini (Muridae, Rodentia). Chromosome Res.

[B4] Chevret P, Dobigny G (2005). Systematics and evolution of the subfamily Gerbillinae (Mammalia, Rodentia, Muridae). Mol Phylogenet Evol.

[B5] Chimimba CT, Bennett NC, Skinner JD, Chimimba CT (2005). Order Rodentia. The Mammals of the Southern African Subregion.

[B6] Colangelo P, Civitelli MV, Capanna E (2001). Morphology and chromosomes of *Tatera* Lataste 1882 (Rodentia Muridae Gerbillinae) in West Africa. Trop Zool.

[B7] Colangelo P, Corti M, Verheyen E, Annesi F, Oguge N (2005). Mitochondrial phylogeny reveals differential modes of chromosome evolution in the genus *Tatera* (Rodentia: Gerbillinae) in Africa. Mol Phylogenet Evol.

[B8] Colangelo P, Granjon L, Taylor PJ, Corti M (2007). Evolutionary systematics in African gerbilline rodents of the genus *Gerbilliscus*: inference from mitochondrial genes. Mol Phylogenet Evol.

[B9] Dobigny G, Aniskin V, Volobouev V (2002). Explosive chromosome evolution and speciation in the gerbil genus *Taterillus* (Rodentia, Gerbillinae): a case of two new cryptic species. Cytogenet Genome Res.

[B10] Dobigny G, Aniskin V, Granjon L, Cornette R, Volobouev V (2005). Recent radiation in West African *Taterillus* (Rodentia, Gerbillinae): the concerted role of chromosome and climatic changes. Heredity.

[B11] Granjon L, Colangelo P, Tatard C, Colyn M, Dobigny G, Nicolas V (2012). Intrageneric relationships within *Gerbilliscus* (Rodentia, Muridae, Gerbillinae), with characterization of an additional West African species. Zootaxa.

[B12] Griffin M, Seely MK (1990). A review of taxonomy and ecology of gerbilline rodents of the Central Namib Desert, with keys to the species (Rodentia: Muridae). Namib Ecology: 25 Years of Namib Research.

[B13] Kovalskaya YM, Aniskin VM, Bogomolov PL, Surov AV, Tikhonov IA (2011). Karyotype reorganisation in the *subtilis* group of birch mice (Rodentia, Dipodidae, *Sicista*): unexpected taxonomic diversity within a limited distribution. Cytogenet Genome Res.

[B14] Musser GG, Carleton MD, Wilson DE, Reeder DM (2005). Superfamily Muroidea. Mammal Species of the World. A Taxonomic and Geographic Reference.

[B15] Pavlinov IJA, Denys C, Granjon L, Poulet A (2001). Current concepts of gerbillid phylogeny and classification. African Small Mammals.

[B16] Perrin MR, Dempster ER, Downs CT (1999a). Gerbillurus paeba. Mamm Species.

[B17] Perrin MR, Dempster ER, Downs CT (1999b). Gerbillurus tytonis. Mamm Species.

[B18] Qumsiyeh MB Chromosomal Evolution in the Rodent Family Gerbillidae. PhD thesis, Texas Tech University, Texas (1986a).

[B19] Qumsiyeh MB (1986b). Phylogenetic studies of the rodent family Gerbillidae: I. Chromosomal evolution in the Southern African complex. J Mamm.

[B20] Qumsiyeh MB (1988). Pattern of heterochromatic variation and phylogeny in the rodent family Gerbillidae. Texas J Sci.

[B21] Qumsiyeh MB, Chesser RK (1988). Rates of protein, chromosome and morphological evolution in four genera of Rhombomyine gerbils. Biochem Syst Ecol.

[B22] Qumsiyeh MB, Hamilton MJ, Schlitter DA (1987). Problems in using Robertsonian rearrangements in determining monophyly: examples from the genera *Tatera* and *Gerbillurus*. Cytogenet Cell Genet.

[B23] Qumsiyeh MB, Hamilton MJ, Dempster ER, Baker RJ (1991). Cytogenetics and systematics of the rodent genus *Gerbillurus*. J Mamm.

[B24] Rens W, Fu B, O'Brien PCM, Ferguson-Smith M (2006). Cross-species chromosome painting. Nat Protoc.

[B25] Robinson TJ, Ropiquet A (2011). Examination of hemiplasy, homoplasy and phylogenetic discordance in chromosomal evolution of the Bovidae. Syst Biol.

[B26] Robinson TJ, Ruiz-Herrera A, Avise JC (2008). Hemiplasy and homoplasy in the karyotypic phylogenies of mammals. Proc Natl Acad Sci USA.

[B27] Rokas A, Holland PWH (2000). Rare genomic changes as a tool for phylogenetics. Trend Ecol Evol.

[B28] Sannier J, Gerbault-Seureau M, Dutrillaux B, Richard FA (2011). Conserved although very different karyotypes in Gliridae and Sciuridae and their contribution to chromosome signatures in Glires. Cytogenet Genome Res.

[B29] Seabright M (1971). A rapid banding technique for human chromosomes. Lancet.

[B30] Sumner AT (1972). A simple technique for demonstrating centromeric heterochromatin. Exp Cell Res.

[B31] Telenius H, Carter NP, Bebb CE, Nordenskjöld M, Ponder BAJ, Tunnacliffe A (1992). Degenerate oligonucleotide-primed PCR: general amplification of target DNA by a single degenerate primer. Genomics.

[B32] Volobouev V, Aniskin VM, Sicard B, Dobigny G, Granjon L (2007). Systematics and phylogeny of West African gerbils of the genus *Gerbilliscus* (Muridae: Gerbillidae) inferred from comparative G- and C-banding chromosome analyses. Cytogenet Genome Res.

[B33] Yang F, Graphodatsky AS, Liehr T (2009). Animal probes and Zoo-FISH. Fluorescence in situ Hybridization (FISH) Application Guide.

[B34] Yang F, Trifonov V, Ng BL, Kosyakova N, Carter NP, Liehr T (2009). Generation of paint probes by flow-sorted and microdissected chromosomes. Fluorescence in situ Hybridization (FISH) Application Guide.

