# Supplementary material for: Tracking Chromosome Evolution in Southern African Gerbils Using Flow-Sorted Chromosome Paints
Source: Cytogenet Genome Res. 2013 May 4;139(4):267–75. doi: 10.1159/000350696 (PMC3721133; doi:10.1159/000350696)
Supplement: Supplementary file 1 — Supplemental Figure [file cgr-0139-0267-s01.pdf]

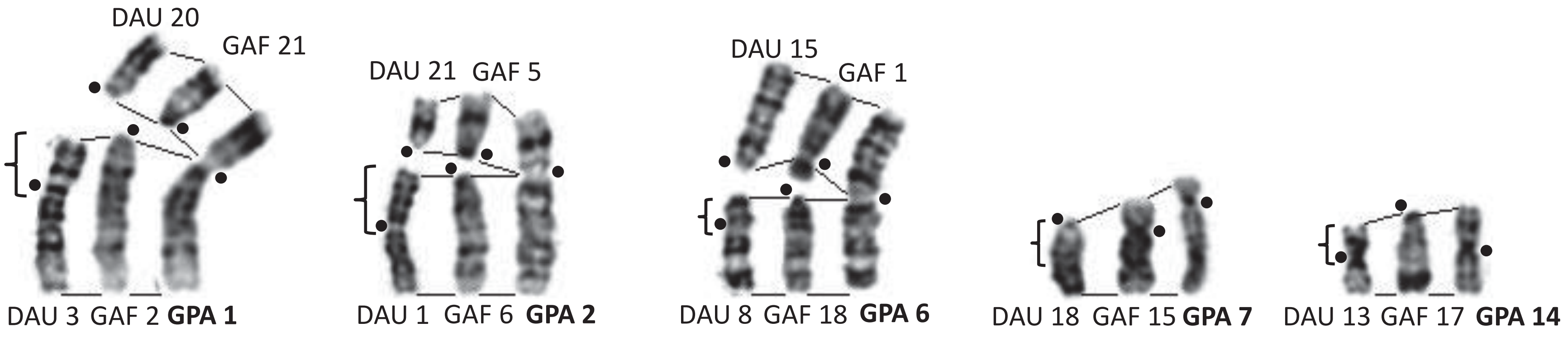
DAU 20
GAF 21
DAU 21
GAF 5
DAU 15
GAF 1
DAU 3 GAF 2 **GPA 1**
DAU 1 GAF 6 **GPA 2**
DAU 8 GAF 18 **GPA 6**
DAU 18 GAF 15 **GPA 7**
DAU 13 GAF 17 **GPA 14**

Suppl. Fig. 1. Partial autosomal G-band comparison directed by Zoo-FISH among *D. auricularis, G. afra* and *G. paeba.* Painting probes GPA1, 2, 6, 7 and 14 indicate that in addition to Robertsonian fusions, a total of 5 inversions were involved during speciation of these rodents. Brackets show the extent of these inversions while dots indicate the position of the centromeres.
